# Recent Trends in Monitoring of European Water Framework Directive Priority Substances Using Micro-Sensors: A 2007–2009 Review

**DOI:** 10.3390/s100907947

**Published:** 2010-08-26

**Authors:** Philippe Namour, Mathieu Lepot, Nicole Jaffrezic-Renault

**Affiliations:** 1 Université de Lyon, Laboratory of Analytical Sciences, UMR CNRS 5180, 43 boulevard 11 novembre 1918, F-69622, Villeurbanne cedex, France; E-Mail: nicole.jaffrezic@univ-lyon1.fr (N.J.-R); 2 Cemagref, UR MALY, CP 220, F-69336, Lyon cedex 09, France; 3 Université de Lyon, INSA Lyon, LGCIE, 34 Avenue des arts, F-69621 Villeurbanne Cedex, France; E-Mail: mathieu.lepot@insa-lyon.fr (M.L.)

**Keywords:** sensors, water framework directive, metals, priority substance

## Abstract

This review discusses from a critical perspective the development of new sensors for the measurement of priority pollutants targeted in the E.U. Water Framework Directive. Significant advances are reported in the paper and their advantages and limitations are also discussed. Future perspectives in this area are also pointed out in the conclusions. This review covers publications appeared since December 2006 (the publication date of the Swift report). Among priority substances, sensors for monitoring the four WFD metals represent 81% of published papers. None of analyzed publications present a micro-sensor totally validated in laboratory, ready for tests under real conditions in the field. The researches are mainly focused on the sensing part of the micro-sensors. Nevertheless, the main factor limiting micro-sensor applications in the environment is the ruggedness of the receptor towards environmental conditions. This point constitutes the first technological obstacle to be overcome for any long-term field tests.

## 1. Introduction

The Water Framework Directive (WFD) governs European water policy. WFD has been in place as the main European regulation for the protection of water resources and the water environment since 2000 [1]. One of its principal objectives is to achieve good chemical and ecological status and to restore water bodies to a ‘good status’ by 2015. WFD requires management of water bodies so that the water quality does not affect their ecological services. Chemical status refers to specific pollutants (e.g., priority substances or priority hazardous substances) for which environmental quality standards (EQS) are proposed and defined for pollutants as minimum requirements [2].

As the WFD implementation gradually comes into effect in European countries, the environmental metrology market is bound to increase over the coming years. In view of the high cost of the laboratory analyses required and the potential artifacts that may be introduced during the conventional sampling protocol: “send a technician, take an isolated sample, send it to the laboratory and analyze it” with at best, in conventional water chemistry, a 24-hours flow proportional composite sample, a new type of field analysis must be designed. An average sample obscures the essentially dynamic character of a polluting event and average contents are devoid of any ecological realism. Biocenoses in rivers are never exposed to average contents, which have no actual existence as far as they are concerned. They are in reality exposed to changes in their physicochemical ambience. A first monitoring of dissolved copper, lead and cadmium with a submersible voltametric flow cell for periods of four days in coastal waters shows that the potentially most toxic forms of metals may vary in concentration on a time scale of less than one hour [3]. This first data confirm the poor ecological relevance of the conventional sampling protocol and the necessity of a continuous measurement. The greater and more sudden these changes, the more disturbance they cause. In terms of toxicology, fluctuation is a more important parameter than the average level, and in the present case the concentration peak reached by the pollutant is more important than the average concentration. The ecological pertinence of such a procedure is more than problematic [4]. New environmental monitoring strategies must be designed. A possible avenue that merits further exploration involves the deployment of low-cost instrumentation allowing massive data logging, as well as tools for subsequent data validation, management and interpretation. However, while this new type of instrumentation is possible, and even desirable, such deployment cannot at the present time cover all the WFD’s parameters and there is little probability of this situation changing before 2015, or even 2021. Consequently, faced with the magnitude of this metrological challenge and the urgency of the situation, a paradigm shift is required in order to imagine a new approach to the problem of water monitoring. Given this situation, current research on micro-sensors is leading to the emergence of many measuring principles. The Swift report (http://www.swift-wfd.com), published in December 2006, lists a wide range of monitoring methods currently available or under development for supporting the WFD. This review covers publications after December 2006, the publication date of the Swift report. It then covers the period extending from January, 2007 to December 2009, and concerns micro-sensors of so called priority substances from the list quoted in appendix II of the Common Decision nº3/2008 of December 20th, 2007 [6]. This review, although not being able to aspire to the exhaustiveness, constitutes a relatively exact panorama of the recent research efforts in the field of the measurement of priority substances in the water using micro-sensors.

For practical reasons, with the analyte constituting the access key for chemical analysts, micro-sensors were firstly classified according to the measured analyte and then, for one analyte, according to the measurement principle or transduction mode: electrochemical and optical. The papers will then be presented according to the following classification: first the measured analyte then, when several technologies are used, they were grouped by type. Finally we tried to focus this review mainly on the micro-sensors at the field validation stage, that is the systems having passed lab tests and susceptible to field validation. The criteria of publication analysis are: the type of aquatic environment studied, the detection limit and the reproducibility.

Finally, with a concern for sustainable development and the eco-design of instruments installed in natural environment, this review does not take into account publications presenting methods involving toxic compounds, such as “heavy metals”, in particular mercury impregnation or films, even if the quantities used are relatively low. Indeed, substances such as cadmium or mercury have been classified as ‘priority hazardous substances’ in the Decision nº2455/2001/EC [5] and Directive 2008/32/CE [6], for which Member States should implement necessary measures with the aim of ceasing or phasing out emissions, discharges and losses into water of those priority hazardous substances which derive from human activities. So it is preferable to banish these hazardous substances from our devices, rather than quibble over low or negligible implemented quantities, and to be vigilant about the potential toxicity of any new substances used in our devices.

Furthermore, development of new devices using priority hazardous substances leads to a commercial dead-end, and a waste of time and money: they cannot be used in Europe, and even in the other parts of the world. Indeed, mercury is recognized as a chemical of global concern. US EPA’s Roadmap for Mercury (5 July 2006) promotes reducing mercury in processes and products, even where cost-effective substitutes do not exist. The overall goal of the Global Mercury Partnership of United Nations Environment Programme (Governing Council Decision 25/5, Nairobi, Kenya, 16–20 February 2009) is to reduce and eventually eliminate mercury use in products and processes, and raising awareness of mercury-free alternatives. Among these products electric and electronic devices are targeted.

## 2. Reviews & Books

Some recent reviews were published during the years 2007–2009. Johnson *et al*. [7] refocus problems posed by the punctual sampling of dynamic systems such as rivers. The authors insist on the necessity of high frequency and continuous measures in time, as well as on the obligation of taking into account spatial variations. They also regret the current absence, for the most part, of simple, selective and sensitive enough sensors for the chemical elements of interest to the geochemist operating without drift over long periods. Pejcic *et al.* [8] surveyed some of the work that has been undertaken using sensors to detect hydrocarbons. Of the various transducers examined in this review the optical-based sensor appears to be the most promising in terms of water monitoring. The Bosch *et al*. review [9] in the journal *Sensors*, is about the use of optical fibre micro-biosensors, mainly in biological applications. Let us note a section dedicated to the measurement of metals via cells or immobilized enzymes and micro-biosensors detecting PCB or pesticides via antibodies immobilized on silica fibres. Also let us quote the Lieberzeit and Dickert review [10] dedicated to the sensors applicable to the environmental chemistry, operational in the field, during these last five years. The authors end their paper with the very scarce literature published on actually operational sensors, albeit with some breakthroughs in the metal measurement domain. The development of simple and strong optical or electrochemical prototypes adapted to the field conditions, will permit one to envisage actual field monitoring in a near future. Clare Reimers’ review [11] is more particularly centred on the usage of glass microelectrodes in oceanography. A section is dedicated to the study of metal speciation in the marine environment with minimal sampling artefacts.

Two articles review the challenges associated with metal speciation studies, and focus on voltammetric techniques for the *in situ* study of metal speciation. More specifically, they summarize the specific conceptual, analytical, and technical criteria that must be considered to develop rugged, field deployable, non-perturbing sensors and probes [12,13]. Lange *et al.* [14] overviewed surface acoustic wave-based biosensor technology, and a small section was devoted to atrazine and PAH detection. The review of Moreno-Garrido [15] draws a general picture of the advances in immobilization techniques and biotechnology, using freshwater and marine micro-algae, in environmental aquatic research for the toxicity measurement of substance or effluent. Jones and Compton’s review [16] summarizes works using boron-doped diamond (BDD) electrodes for stripping voltammetry, particularly for measurements of toxic metals such as cadmium or lead. BDD does not seem to be a suitable mercury substitute in all cases, but is one of the more widely applicable alternative electrode materials in comparison to glassy carbon or solid metal electrodes. Jaffrezic-Renault and Dzyadevych [17] presented the application of the conductometric measurement method to environmental monitoring and specifications obtained for the detection of different pesticides, herbicides and heavy metal ions, based on enzyme inhibition.

In a detailed review, Seidel and Niessner [18] described microarray techniques for the simultaneous detection of multiple analytes and presented some environmental applications such as pesticides, toxins and endocrine-disrupting compound detection in water. Recent advances in the development and applications of nucleic acid-based biosensors for environmental application are reviewed by Palchetti and Mascini [19], with special emphasis on functional nucleic acid elements and the detection of chemically-induced DNA damage caused by genotoxic pollutants as some PAH, pesticides or solvents. A chapter of book “*Antiterrorism and Homeland Defense: Polymers and Materials*” is devoted to porphyrin-enzyme complexes for the detection of organophosphates using evanescent wave absorbance spectroscopy (EWAS) [20]. Nolan and Lippard [21] provided a very comprehensive account of progress in the design and application of optical sensors for mercury, in the environmental and biological media, up until July 2007.

Diamond *et al*. [22] present the concept of ‘wireless sensor networks’. A part of their review is dedicated to remote environmental monitoring and highlights limitations with the current manifestations of these platforms, particularly with respect to integration of chemo-/biosensing capabilities. In the same way, Strobl and Robillard [23] give insight into the strategies of water quality monitoring network design for surface freshwaters, along with their weaknesses and shortcomings. They propose nine recommendations for the successful development of a methodology for designing monitoring networks. A section of Bogues’ article [24] gives a brief overview of the environmental sensing technologies in water quality monitoring and considers briefly the research effort.

Jaffrezic-Renault [25] presents in *Actualité Chimique* different kinds of electrochemical micro-sensors allowing the *in situ* monitoring of pollutants in waters: heavy metals, ammonium, total organic carbon, organophosphate pesticides. The review of Baruah and Dutta [26] on nanotechnology applications in pollution sensing in agriculture and environment contains an important section on the measurement of pollutants by means of nano-structured materials. This review is far from exhaustive concerning the topic of micro-sensors and certain references are more than 10 years old. Pesavento *et al*., [27] presented the electrochemical methods for metal speciation in natural waters, mainly by means of voltammetry. It is noteworthy that a section is dedicated to *in situ* measurements, especially focused on practical needs. The single micro-sensor presented is a gel electrode containing mercury. The second part of this review deals with alternative procedures as separation techniques based on ion exchange and complexing resins, and micro separation methods as Donnan membrane technique, diffusive gradients in thin-film gels and permeation liquid membrane. Reardon *et al*.’s review [28] focuses on photoluminescence-based biosensors of potential use in the environmental applications. They give examples of optical biosensors for organics (aromatic solvents, PAH, organophosphate or halogenated pesticides) and metal determinations (Hg, Cd, Pb and Ni), however these references are previous to 2007. Leray and Value [29], after a summary of the main classes of fluorescence molecular receptors for the detection of toxic metal ions (Photoinduced Electron or Charge Transfer, Excimer Formation or Disappearance and Energy Transfer), present different calixarene-based fluoroionophores for the detection of caesium, mercury, lead and cadmium. A review of Selid *et al*. [30] examines different useful micro- and nano-sensors for mercury determination, mainly in healthcare and in a lesser extent, in the environmental field. Finally, David *et al*. [31] published a review on alkylphenols in marine and coastal environment, mainly centred on the environment contamination, however a section is dedicated to their detection in environmental matrices by biological tests on cells but not strictly involving micro-sensors as we understand them.

## 3. Scientific Articles

### 3.1. Metals

The WFD targets four priority metals: cadmium, mercury, nickel and lead [1]. Table 1 summarizes the various target values for freshwater water bodies. The strongest constraints are given by the SEQ-Eau, a water quality evaluation system developed by French Water Agencies in order to maintain the water biological balances in an optimal way. The detection limits of micro-sensors should be equal or lower than SEQ-Eau target values, although they do not represent statutory values, as the limit values of the Order 2001-1220 are only imperative for drinking water.

The analysis of the publications from 2007 to 2009 in the field of micro-sensors, shows that research efforts mainly concern the determination of WFD metals in waters (126 papers), the detection of WFD organic substances being the subject of less attention (24 papers), then 83% of the papers concern only 17% of priority substances. The sensors for metal detection divide up roughly equally between electrochemical (56%) and optical (44%) techniques, with strong disparities according to the metal concerned. As a general rule the electrochemical methods are preferred for the detection in water of cadmium (86%), nickel (67%) and lead (86%). On the other hand optical methods are proposed for mercury detection in 73% of the papers.

Approximately twenty articles deal with the continuous determination of cadmium in water, mainly (81%) by electrochemical techniques (Table 2). Only five articles about optical techniques for cadmium determination were published from 2007 to 2009 (Table 3).

Around 50% of electrochemical sensor display a detection limit for cadmium below 1 μg/L [34–41] on modified carbon electrodes. Other micro-sensors give detection limits ranging from 1 μg/L [42–46] to 100–120 μg/L [60] and 500 μg/L [62] on enzymatic biosensors, 3.6 μg/L using anodic stripping voltammetry on screen printed electrodes doped with bismuth nanoparticles [54] and 20 μg/L by differential pulse voltammetry through polyvinylchloride-2-nitrophenylethyl ether gel [58].

Optical sensors are two or three magnitude order less sensitive than electrochemical sensors. The most sensitive optical sensor displays a detection limit equal to 60 μg/L [63], then are following: 130 μg/L for fluorescent sensor [64] and 560 μg/L for the virtual sensor [65].

Even the detection limits of electrochemical sensors are still too high for water monitoring. The EQS values recommended by the WFD in freshwater range from 0.08 μg/L to 0.25 μg/L according to water hardness; INERIS publishes for cadmium, a PNEC equal to 0.21 μg/L [68] and the SEQ-Eau target value for an optimal water quality goes from 1 ng/L to 9 ng/L, according to water hardness [32]. Finally none of these micro-sensors was validated in a natural environment.

#### 3.1.1. Mercury

Contrary to cadmium determination, for which electrochemistry is widely used, researchers seem to have preferred optical methods for mercury determination in water. Fifty-five articles deal with micro-sensors measuring mercury in water:

- Forty-three articles suggest measuring mercury by an optical means: molecular absorption (Table 4); and fluorescence (Table 5) or by chemiluminescence (two sensors). Among the optical methods, let us note the indirect method of Dittman *et al*. [69] based on the correlation between 254 nm absorbance of hydrophobic acid fraction of dissolved organic matter and mercury concentration. This method was successfully applied to waters of three different forested watersheds in New Hampshire, Vermont, and the State of New York (USA). It gives determination coefficients equal to, respectively, 0.96, 0.99 and 0.98 and its detection limit would be around 0.5 ng/L, corresponding to 0.05 absorbance unit. The authors attribute this correlation to the high content of reduced sulphur sites in hydrophobic acid fraction of organic matter;
- Fifteen articles propose an electrochemical method (Table 6).

Among the mixed optical methods, let us point out two techniques:

- Lee and Mirkin’s method, associating DNA arrays using DNA-modified gold nanoparticle probes and a conventional flatbed scanner to measure scattered light, after silver amplification, gives a detection limit of 2 μg/L [70];
- The virtual sensor of Wang *et al.* [65] combining together nine cross-reactive sensing fluorescent elements on micro-plate with pattern recognition software in order to discriminate and quantify Hg in water. The sensor arrays are excited with a broadband UV lamp and four filters are used for emission detection: 380–500 nm λ_max_= 435 nm; 480–600 nm λ_max_ = 525 nm; 523 nm and 580 nm. The array capacity was tested at different ranges of pH and at different cation concentrations using linear discriminant analysis. Quantitative analysis can be achieved with 93% accuracy in the concentration ≥20 mg/L [65].

Two sensors are based on luminescence, the first one is based on bioluminescence on recombinant *E. Coli* immobilised on optical fibres [109]; and the second one is based on chemoluminescence of an iminophenol trimers [110]. Their detection limits are respectively 2.6 μg/L and 10 μg/L.

If we put aside the detection limit of Dittman *et al*.’s method [69] which would demand a validation on waters different to waters from forested watersheds (nitrate interference), the detection limits of the majority of these micro-sensors are still too high for an environmental application, but not too far from the “0.05 μg/L target”. Certain optical micro-sensors reach the detection limit of 0.2 μg/L by spectrometry [71,72,74] or fluorimetry [87,88], and electrochemical micro-sensors come down to the limit detection of 0.1 μg/L [38,111].

Only two micro-sensors reach the “0.05 μg/L target”, the first one with a detection limit 0.002 μg/L at pH 1 on diamond like carbon electrode [112] and the second one with a detection limit 0.03 μg/L but on acetate buffer at pH 4.2 on screen printed paste carbon electrode [113]. These micro-sensors are ready for the targeted or recommended values for water pollution control: WFD fixes a target value at 0.05 μg/L, but they are still no enough sensitive for reaching the SEQ-Eau target value: 0.007 μg/L [32]. Testing of these sensors for *in situ* mercury determination still remains to be done. None of these micro-sensors was evaluated in the field; only Dittman *et al*.’s method [69] should be applicable in the field with a commercial UV probe, of which spectral deconvolution algorithms should be fitted, and with a specific calibration.

#### 3.1.2. Nickel

Only six articles (Table 7), among which two come from the same team, deal with nickel detection. Among these six papers, four use electrochemical methods: cyclic voltammetry [113,124], or conductometry [42,62], and two teams use optical methods – fluorimetry [65] or colorimetry [125].

Electrochemical methods give lower detection limits than optical methods. Only the electrochemical micro-sensors, once validated in laboratory, should be useful for nickel monitoring within the WFD framework (AA-EQS: 20 μg/L). Only the bismuth-film electrode micro-sensor [124] covers completely the INERIS or SEQ-Eau requirements, their target values being, respectively, 0.5 μg/L and 0.25 μg/L.

#### 3.1.3. Lead

Thirty-six articles describe micro-sensors measuring lead in waters, the majority of them propose electrochemical methods (Table 8), only five articles present optical methods. The majority of the published electrochemical methods are voltammetric methods, mainly on modified carbon electrodes (paste or glassy carbon, graphite) with detection limits below the SEQ-Eau target value: 0.21 μg/L [34,40,126,127,128]. Turek *et al*., develop a virtual sensor founded on fuzzy logic data analysis of combined potentiometric signals from Ag-, Cu- and Pb-selective chalcogenide glass sensors (AgIAsSe, CuAgAsSe, PbI_2_Ag_2_SAs_2_S_3_). This method recognizes an unknown metal in solutions with 100% probability, for heavy metal concentrations higher than 210 μg/L [129].

Predicted Non Effect Concentration (PNEC) for lead, advocated by INERIS (PNEC: 5 μg/L), is only achieved using voltammetric sensors ([35,39,43,44,49,51,52,54,130–132] and one conductometric biosensor based on *Chlorella vulgaris* [42]. However, this sensor shows a poor specificity. Indeed, heavy-metal ions acting as algal alkaline phosphatase inhibitors, so cadmium, cobalt, nickel and lead exhibit the same detection limit (1 μg/L).

Finally among published optical methods (Table 9), the most sensitive micro-sensor uses DNA probes and fluorescence quenching or turn-off. Pb(II) is measured by ADNzyme activation and DNA cleavage of two fluorescent cyanine probes: Cy3 (550/570 nm) for the detection and Cy5 (650/670 nm) for the reference [135]. Liu *et al.* [90] use a thrombin-binding aptamer probe labelled with donor carboxyfluorescein and quencher 4-([4-(dimethylamino)phenyl]azo)benzoic acid at its 5′ and 3′ termini, respectively. These two optical methods give acceptable detection limits for water monitoring, with respectively: 2 μg/L [135] and 0.1 μg/L [90]. Two other optical methods are colorimetric ones and present too high detection limits: 18 μg/L [136] and 1.3 mg/L [138].

All the aforementioned sensors are adapted to lead monitoring in water according to the WFD criterion: target value at 7 μg/L. However none of these micro-sensors is still at the evaluation stage and ruggedness and hardiness will have to guide selection of the micro-sensors fit for field validation.

#### 3.1.4. Conclusions on metal determination

Mainly because of environmental regulations, the current research on sensors for metal determinations is under a kind of double bind: mercury elimination from devices and keeping the detection limits of mercury electrodes. This particularly stimulating situation will lead to new greener micro-sensors. These new generations of sensors are still in their infancy and many approaches are being explored. The electrochemical approach is the privileged technique by the four fifths of the authors, at least for Cd, Ni and Pb. For the mercury titration there is an inverse proportion with dominance in 80% of the optical approach. It is worthy noting that representing about 60% of published papers, the research efforts in the WFD metal determination seem at present to be to concentrate on mercury detection. The activity seems more particularly active in the research for new chromophores more specific and more sensitive, and of original couplings between chromophore and ionophore. Finally, some articles investigate the potential of virtual sensors, otherwise known as “soft sensors” or “smart sensors”, in heavy metal monitoring. These sensors consist of an array of simple and reliable sensors that are not analyte-specific but can be linked by a computer programmed to process certain sample features and build a proxy of the “unsensed” metal [65,129]. Multivariate calibration, a statistically inspired modification of partial least square method, is applied to DPASV voltammograms acquired on carbon screen-printed electrodes. This data processing leads to better overall root mean square error of prediction values for Cd and Pb. The authors propose to investigate the application of artificial neural networks to the voltammograms in order to reduce the errors and to improve the number of target metals [139]. Until now, their detection limits are too high, and research should be carried out in order to make them operational tools in the field.

## 4. Organic Substances

Among priority substances listed in Appendix II of the Common Decision taken on 20 December, 2007 by the Council of Europe [2], 36 are organic substances, the five remaining being metals (Cd, Ni, Pb and Hg) and an organotin. Organic substances can be grouped in main chemical or usage groups: 17 pesticides, 10 chlorinated solvents, eight PAH and miscellaneous organic substances as alkylphenols, detergents, brominated diphenyl ether, aryl-halides and benzene. Table 10 summarises the superior limit concentrations recommended or statutory in freshwater.

However the scientific publications concerning priority WFD organic substance determinations by means of micro-sensors represent a more modest proportion, than that dedicated to four WFD metals. During years 2007–2009 we counted only two dozens of papers, mainly on detection of organophosphates and of some carbamates (Table 11). Among these, twelve articles present electrochemical methods on modified electrodes, among which seven enzymatic biosensors based on choline esterase activity. Other micro-sensors are intended to measure atrazine [140–142], isoproturon [143], paraquat [144] or DDT-like compounds [145]. Three articles propose sensors for solvent determination in water: two based on infrared spectroscopy and one on impedance spectrometry.

### 4.1. Organophosphates and Carbamates

Among organophosphate pesticides (Table 11) only chlorpyrifos appears among the thirty-three WFD substances, with an AA-EQS of 0.03 μg/L. However the proposed colorimetric method on gold nanoparticles presents high detection limits of several orders of magnitude for the titration of chlorpyrifos and malathion (20 μg/L and 100 μg/L, respectively). [146]. Acetylcholine esterase activity monitoring methods using voltammetry developed by Zhao *et al*., [147] and by Yin *et al*., [148] allow to reach lower concentrations, respectively: 0.5 ng/L for paraoxon and 1 ng/L for parathion [147], 6 ng/L for paraoxon methyl and 7 ng/L for carbofuran [148]. These methods would deserve to be tested on both WFD organophosphates: chlorpyrifos and chlorfenvinphos. Nevertheless these biosensors determine the total concentration of organophosphates and carbamate pesticides, and not a specific compound. Now, the definition of good chemical status in the WFD imposes the measurement of priority organic substance concentrations and not the measurement of toxic effects.

### 4.2. Other pesticides

Among other methods proposed for pesticide determination (Table 12), three methods are proposed for triazine measurement. Pardieu *et al*. [140] presented an electrochemical micro-sensor, based on a molecularly imprinted conducting polymer, deposited on a platinum electrode, able to detect, by cyclic voltammetry, triazines (atrazine, terbutylazine and simazine) and to measure atrazine with a detection limit of 4.5 μg/L. An immunoassay based on a commercially available surface plasmon resonance (SPR) biosensor was proposed. Measurement can be performed in 25 minutes, including the regeneration cycle, and its detection limit in laboratory is 20 ng/L and 26 ng/L in a river sample [141]. A microbioreactor and integrated sensors for the online measurement of oxygen production was used to measure benzalkonium chloride and atrazine effects on the macrophyte *Elodea canadensis*. No observable adverse effect concentration (NOAEC) for atrazine is 10 μg/L [142]. Gouzy *et al*. [143] developed an immunosensor based on SPR for the measurement of isoproturon. The rat monoclonal anti-isoproturon antibody was reversibly immobilized through the use of a capture mouse anti-rat monoclonal antibody, which was covalently immobilized on the sensor chip surface. The detection limit could have reach 0.1 μg/L, but the operational range goes from 1.3 μg/L to 16.3 μg/L (inhibition from 20 to 80%). Lee *et al* [144] measured morphology and resonance characteristics of human hepatoma cell line cultured onto an indium tin oxide layer on the surface of a quartz crystal modified with a collagen film, for the study of the effect of paraquat (2.7 μg/μL) through resonance frequency and resonance resistance responses. The system gives a qualitative response and allows the visualization of the action of paraquat on cells. Finally, an immunosensor based on commercially available SPR has been developed for the monitoring of DDT, its metabolites and analogues in real water samples. Low detection limits are attained for DDT-selective: 15 ng/L; and DDT group-selective immunoassays: 31 ng/L [145].

### 4.3. Solvents

The restricted number of solvent sensors developed (Table 13) present detection limits higher than the WFD and SEQ-Eau’s target values (Table 10), respectively 10 μg/L and 0.5 μg/L for benzene and 2.5 μg/L and 1.2 μg/L for chloroform.

Finally, Kurup [162] describes a virtual sensor constituted of an array of seven SnO_2_ micro-sensors doped with different impurities (as palladium or platinum) and equipped with a gas sampling membrane and integrated into a probe used for the volatile organic pollutant detection in underground environment. This technology supplies at the moment qualitative results (three levels: low, average and high), which must be then confirmed by conventional analyses in laboratory.

## 5. Conclusions

As noticed above, 81% of the publications appeared in 2007–2009 concern only 12% of the priority substances included in the WFD list, namely metals (e.g., Cd, Hg, Ni, Pb). The origin of this disparity already noticed in the production is doubtlessly attributable to the heterogeneousness of chemical structures listed in WFD list which exceeds the financial capacity for sensor development or imposes one weakly profitable development of specific micro-sensor for each priority substance. The fact that only the organophosphates and carbamates are subject of a relatively important number of publications among those dedicated to organic substances (57%), tends to confirm this origin. Indeed these sensors are based on acetylcholinesterase inhibition by organophosphate and carbamates. So, the conception of micro-sensors measuring biological effects rather than concentrations could constitute an innovative way allowing the release from constraint of the development of substance specific micro-sensors. However the notion of “Environmental Quality Standards” in the WFD sense imposes the measurement of specific concentrations and not some effects. This normative aspect of the Directive establishes a strong constraint in the research for new analytical devices.

None of articles published during 2007–2009 presents a micro-sensor totally validated in the laboratory and ready for tests under real field conditions. Research is mainly focused on the sensitive part of the micro-sensors: the receptor. Now, even if progress still remains to achieve, in particular to lower the limit of detection, the principles of detection do not seem to constitute the main limiting factor the development of environmental micro-sensors. The sensitivities of the best cadmium or mercury sensors exhibit detection limits respectively five and two folds higher than SQE target values. It is too high for *in situ* measurement in natural waters, but these sensors could be right now used for the industrial wastewater control (e.g., electroplating plants). During the wait for a better sensitivity, these mercury-free sensors could be associated to concentration devices in lab-on-a-chips, in order to lower the detection limits.

On the other hand, receptor ruggedness to environmental conditions must be improved, in particular concerning their fouling resistance; technologies such as surface passivation, or ultrasound cleaning of the sensitive surface, should allow progress in this domain. This point constitutes the first technological obstacle to be overcome, before going into long-term tests in real environments.

Other scientific and technological obstacles to overcome in order to build operational micro-sensor networks are at the transducer and the transmitter levels. Setup up a micro-sensor in the natural environment requires an infrastructure capable of fuelling it with energy, watching it and collecting data. This means that skills outside the fields of chemistry, such as physics, electronics and computing, which will be required in order to build a complete *in situ* measurement chain. The main axes of progress must be centred on the following points:

– Miniaturization: pursue the integration of the various modules (receptor, transducer and transmitter) on the same chip. It leads to a decrease of the energy consumption and seems to have, at least on the conductometric electrodes, a beneficial effect on the sensitivity. This miniaturization requires for skills in micro-technology;
– Communication: assure the communication between the micro-sensors and the readout station and between the micro-sensors. The network should have the possibility of positioning these sensors (search for micro-sensors scattered after a flood in particular), of verifying the state of its communications with the readout station and its neighbour micro-sensors, of validating the quality of its data before their transmission, of alerting in case of abnormal situation. Two wireless communication modes should be studying: ultrasound-based or radio frequencies;
– Autonomy: decrease the energy consumption or develop possibilities of *in situ* energy supply. Sedimentary microbial fuel cells or marine turbines would be technologies that should be investigated;
– Eco-design: integrate the possible impact of the chosen technology on the environment, as well as life cycle of the micro-sensor, and principles of measurement. Indeed in the eventuality of a loss of the material in the environment, the micro-sensor composition has to banish the use of well-known toxic or dangerous matter for the environment. This point concerns all devices introduced into the environment: we have to minimize as much as possible any toxic impact of our devices. Indeed, the WFD [1] demands, for priority hazardous substances from human activities (as cadmium or mercury), the cessation or phasing out of discharges, emissions and losses into water before 2020. This regulation coming into force in the Member States, the use of mercury is banished in the short-term. Also, development of new devices using priority hazardous substances leads to a commercial dead-end, and a waste of time and money: it cannot be used in Europe. Here and now, the regulatory requirements must be included in the design of new monitoring devices and we have to minimise the potential toxic impact of all components used to build efficient monitoring sensor network platform. Clearly, there is plenty of room for progression.

Finally it is advisable to mention the necessity of developing data processing technologies to manage in an optimal way the considerable data flows produced by a micro-sensor network. There are very significant challenges for the micro-sensor research community for delivering sensing platforms that are appropriate for integration into scaled-up deployments in terms of sustainability, cost, and reliability. Calls for proposals should centre on the conception of complete micro-sensor networks (material and software) and on the emergence of projects having as a global objective an operational monitoring network, from the receptors to the management computer.

## Figures and Tables

**Table 1.** Summary of the superior limit concentrations (μg/L) of metallic elements, recommended or statutory in fresh water.

	**Surface water (good chemical status)**			**Drinking water**	
	WFD [2] AA-EQS	INERIS** PNEC	SEQ-Eau [32]	Order in Council 2001-1220 [33]	SEQ Underground water
Cd	0.08–0.25	0.21	0.001	5	1
Hg	0.05	0.24	0.007	1	0.5
Ni	20	0.5	0.25	20	10
Pb	7.2	5	0.21	10	5

* AA-EQS: Environmental quality standards expressed as an annual average value;

** INERIS values are PNEC (Previsible Non Effect Concentration) from the following reports: Cd [29]; Hg [30]; Ni [31]; Pb [32]. SEQ-Eau: Système d’Evaluation de la Qualité de l’eau (water quality evaluation system) developed by the French Water Agencies.

**Table 2.** Analytical characteristics of electrochemical sensors for cadmium determination in water, published between 2007 and 2009.

**Method**	**Tested sample**	**LoD (μg/L)**	**RSD (%)**	**Ref.**
DPASV on multiwalled carbon nanotubes/Bi film modified glassy carbon	Tap, river & spring waters	0.2	4.6	[34]
DPASV on boron-doped diamond	Acetate buffer	0.4	nr	[35]
DPASV on bismuth film/graphite electrode	Acetate pH 4	0.5	nr	[36]
SWASV on hydroxyapatite modified carbon paste electrode	Tap water	0.5	3.8	[37]
Inhibition of urease immobilized on screen-printed 10% rhodinized carbon electrode	Standards	0.6	nr	[38]
DPASV on bismuth/poly(p-aminobenzene sulfonic acid) film electrode	Tap water	0.6	3.4	[39]
SWASV on antimony film carbon paste electrode	Spiked lake water	0.8	3.8	[40]
DPASV on multiwalled carbon nanotubes-sodium dodecyl benzene sulfonate modified stannum film electrode	Tap water	0.9	4.5	[41]
Conductometric measurement of alkaline phosphatase activity of the microalgae *Chlorella vulgaris* on two gold interdigitated electrodes	Wastewater	1.0	nr	[42]
SWASV on bismuth-film electrode	River water	1.0	4.4	[43]
LASV on a zeolite NH4-Y modified carbon paste electrode	Ground & wastewaters	1.0	7	[44]
SWASV on polymer-modified glassy carbon electrode	Plating wastewater	1.0	2.5	[45]
Conductometric measurement of alkaline phosphatase activity of the microalgae *Chlorella vulgaris* on two platinum interdigitated electrodes	Tris-HCl buffer	1.0	7	[46, 47]
SWASV on square array of 8 × 8 screen-printed carbon-based microelectrodes	River	1.3	18	[48]
DPASV on glassy carbon electrode modified with a thiacalix[4]arene film	River & lake waters	2.2	3.2	[49]
SWASV on bismuth nanoparticle modified boron doped diamond	Standard pH 1.2	2.3	nr	[50]
SWASV on glassy-carbon electrodes functionalized with composite of Nafion and thiol self-assembled monolayers on mesoporous supports	River water & seawater	2.5	5	[51]
SWASV on screen-printed graphite electrode	Seawater	2.9	2.3	[52]
Cyclic voltammetry of invertase-glucose oxidase activities on platinum microelectrode	Phosphate buffer	3.0	nr	[53]
SWASV on carbon disk screen-printed electrode with Bi ions	Wastewaters Spiked tap water	3.6	10	[54]
SWASV on nitrogen doped diamond-like carbon microelectrode array	Acetate pH 4	4.6	nr	[36]
DPASV on screen printed carbon electrode	Mine water	9	4.5	[55]
ASV on bismuth electrode	Pore & ground water	9.3	2	[56]
ASV on screen-printed bismuth oxide/graphite electrode	Wastewater	16	9.1	[57]
DPASV on tape ion sensor based on metal ion transfer reactions at the water/gel micro-interface	Standards	20	nr	[58]
LSASV on antimony nanoparticle modified boron doped diamond	Standard pH 1	38	nr	[59]
Potentiometry on ion-selective electrodes with PVC/4-hydroxy salophen membrane	River water & wastewater	100	1	[60]
Potentiometry on ion-selective electrodes with PVC/thiacalix[4]arene membranes	River water	120	nr	[61]
Conductometric measurement of alkaline phosphatase activity in enzyme membranes on gold interdigitated electrodes	Tris-nitrate buffer	500	4	[62]

* LoD: Limit of Detection; RSD: Relative Standard Deviation; nr: not reported; DPASV: Differential Pulse Anodic Stripping Voltammetry; SWASV: Square-Wave Anodic Stripping Voltammetry; LSASV: linear-scan anodic stripping voltammetry.

**Table 3.** Analytical characteristics of optical sensors for cadmium determination in water, published between 2007 and 2009.

**Method**	**Tested sample**	**LoD (mg/L)**	**RSD (%)**	**Ref.**
Absorbance of 4-hydroxy salophen on triacetyl cellulose membrane at 431 nm	Wastewater & river water	0.06	2.9	[63]
Fluorescence enhancement of porphyrin-terpyridine complex	River waters	0.13	4.1	[64]
Nine cross-reactive sensing fluorescent elements on micro-plate & data processed by pattern recognition	Mineral waters	0.56	nr	[65]
Photoluminescence enhancement of CdS:Mn/ ZnS Qdots in 1,10-diaza-18-crown-6 shell	Standard	11	nr	[66]
Fluorescence enhancement of 8-hydroxyquinoline (319/410 nm)	Standard	3,000	nr	[67]

* LoD: Limit of Detection; RSD: Relative Standard Deviation; nr: not reported; DPASV: Differential Pulse Anodic Stripping Voltammetry; SWASV: Square-Wave Anodic Stripping Voltammetry; LSASV: linear-scan anodic stripping voltammetry.

**Table 4.** Analytical characteristics of colorimetric sensors for mercury determination in water, published between 2007 and 2009.

**λ_ab_ (nm)**	**Chromophore**	**Tested sample**	**LoD (μg/L)**	**RSD (%)**	**Ref.**
650	2-Mercapto-2-thiazoline and chromo-ionophore/PVC membrane	River waters	0.01	0.76	[71]
580	4-Phenyl-2,6-bis(2,3,5,6-tetrahydrobenzo[b] [1,4,7]-trioxononin-9-yl)pyrylium perchlorate/PVC membrane, (turn-on)	Shaft & fountain waters	0.02	0.04	[72]
663	2-Mercaptopyrimidine in PVC membrane (turn-off)	River waters	0.08	0.42	[73]
405	3,30,5,50-Tetramethylbenzidine on DNA probe (turn off)	Spiked creek waters	0.2	nr	[74]
520	4(2-Pyridylazo)resorcinol	Spiked water	0.22	5	[75]
450	Tetraphenylporphinetetrasulfonic acid on mesoporous monolith	Buffer pH 9	1.2	0.4	[76]
557	Tetrapyridine on “dip-sticks (turn-off)	Standards	5	nr	[77]
400/530	Spiropyran probe (turn on).	Spiked tap water	20	nr	[78]
429	4-Hydroxysalophen	Tap & river water	26	1.8	[79]
665	Hexathiacyclooctadecane	Tap & river water	40	2.4	[80]
642	Squaraine dye derivatives	Standards	100	nr	[81]
560	1-[2-Pyridylazo]-2-naphthol in triacetyl cellulose	River & waste waters	160	3.1	[82]
254	Hydrophobic acid fraction in DOC used as proxy of dissolved Hg	River & lake water	300		[69]
520/300	Naphthalimide derivative on gold nanoparticles	Standard	500	nr	[83]
490	Cyclotriveratrylene derivative	River water	1,000	0.9	[84]
530	Sensitised alumina cladding and PLS model	Standards	1,000		[85]
542	Azo-coupled macrocyclic dye, on a silica nanotube	Standards	20,000	nr	[86]

* LoD: Limit of Detection; RSD: Relative Standard Deviation; nr: not reported.

**Table 5.** Analytical characteristics of fluorimetric sensors for mercury determination in water, published between 2007 and 2009.

**λ_ex_/ λ_em_ (nm)**	**Chromophore**	**Tested sample**	**LoD (μg/L)**	**RSD (%)**	**Ref.**
510/581	Rhodamine on thioglycolic acid modified gold nanoparticles (−)	River water	0.01	1.2	[87]
485/535	Polarization on DNA functionalized gold nanoparticles (+)	Spiked river water	0.2	nr	[88]
500/550	Rhodamine derivatives (+)	Spiked waters	1	nr	[89]
475/510	carboxyfluorescein on 4-([4-(dimethylamino)phenyl]azo) benzoic acid binding on DNA probes	Pond water	1	nr	[90]
401/538	Naphthalimide derivative of 2,6-bis(aminomethyl)pyridine	Tap waters	1.4	2.3	[91]
520/600	Polythymine oligonucleotide T33, citrate-capped gold nanoparticles	Pond water	2	8	[92]
273/519–527	T–T mismatch base pairs, (−)	Spiked river water	4		[93]
425/646–603	Prophirin-quinoline (turn-off at 646 nm and turn on at 603 nm)	Spiked river waters	4.4	nr	[94]
490/510	Oligonucleotides, DNA intercalators, and conjugated polymers	Standards	6.4	nr	[95]
228–280/330	CdS-encapsulated DNA nanocomposite (−)	Wastewater	8		[96]
438/673	Porphyrin/PVC membrane (−)	Spiked river water	8	4	[97]
273/519–527	T–T mismatch base pairs (+)	Buffer	8.4		[98]
480/525	DNA-functionalized gold nanoparticles & OliGreen	Spiked pond water	10		[99]
499/624	Seminaphthofluorescein chromophore. (+)	River waters	10	4.5	[100]
365/488	Coumarinylalkyne (−)	Buffer pH 7.4	20		[101]
458/534	2-[(Aminoethylthio)propylthio]ethanamine on 7-nitro-benzo-2-oxa-1,3-diazolyl moieties (+)	Acetonitrile/water	20		[102]
415/ratio 525/650	Naphthalimide-porphyrin hybrid (+)	River waters	20	4.4	[103]
633/ratio 501/403	Polymer thiourea-thiadiazole-pyridine (+)	Water/Tetrahydrofuran	22.6	nr	[104]
490/591–520	CdSe quantum dots surface-modified with triethanolamine	Standards	36	nr	[105]
352/500	Quinolinocyclodextrin derivative (−)	Standards	60	nr	[106]
246/309	N,N′-(1,4-phenylenedimethylidyne) bis-1,4-benzene-diamine (−)	Ethanol/water 9:1	120	nr	[107]
303/487	8-Benzyloxyquinolinebased ester (−)	Standards	520	nr	[108]
300–400/	Nine cross-reactive sensing fluorescent elements on micro-plate and data processed by pattern recognition (−)	Mineral waters	20,000	nr	[65]

* LoD: Limit of Detection; RSD: Relative Standard Deviation; nr: not reported; (−) turn off, (+) turn on

**Table 6.** Analytical characteristics of electrochemical sensors for mercury determination in water, published between 2007 and 2009.

**Method**	**Tested sample**	**LoD (μg/L)**	**RSD (%)**	**Ref.**
DPASV on Boron-doped diamond like carbon electrode	Standard pH 1	0.002	10	[112]
DPASV on screen-printed carbon paste microelectrode chip	Standard solutions	0.03	nr	[113]
Amperometric measure of ammonia from urease on rhodinized carbon electrode	Standards	0.1	nr	[38]
DPSV on labeled DNA gold nanoparticles	Tap & river waters	0.1	4	[111]
Cyclic voltammetry on invertase-glucose oxidase modified Pt electrode (inhibition)	Spiked waters	2	nr	[53]
Field Effect Transistor on single walled carbon nanotube sensor	Standards	2	nr	[114]
Potentiometry on 2-amino-6-purinethiol or 5-amino-1,3,4-thiadiazole-2-thiol/PVC membrane	Natural waters	8.8	3	[115]
SWASV on 2-aminothiazole modified carbon paste electrode	Tap & waste waters	12	10	[116]
Potentiometry on substituted thiourae-functionalized nanopourous silica	Wastewater	14	4.5	[117]
DPSV on labeled DNA with a ferrocene on a gold electrode surface	Buffers	20	nr	[118]
Potentiometry on bis(benzoyl acetone) diethylene triamine/PVC membrane	Natural & waste waters	20	0.5	[119]
Potentiometry on salophen modified carbon paste electrode	Natural waters	30	0.8	[120]
LASV on Nitrogen doped tetrahedral amorphous carbon thin films/silicone	Standard pH 1	200	nr	[121]
ASV on chitosan modified carbon paste electrode	Spiked waters	130	5	[122]
Chrono-amperometry on glucose oxidase in poly-*o*-phenylenediamine film (inhibition)	Standard	500	6	[123]

* LoD: Limit of Detection; RSD: Relative Standard Deviation; nr: not reported; DPASV: Differential Pulse Anodic Stripping Voltammetry; SWASV: Square-Wave Anodic Stripping Voltammetry; LSASV: linear-scan anodic stripping voltammetry.

**Table 7.** Analytical characteristics of sensors for nickel determination in water published between 2007 and 2009.

**Method**	**Tested sample**	**LoD (μg/L)**	**RSD (%)**	**Ref.**
SWASV on bismuth-film electrode after complexation with dimethylglyoxime	River water	0.1	2.3	[124]
DPASV on carbon screen-printed micro-electrode chip	Acetate buffer	0.5	nr	[113]
Conductometric measurement of alkaline phosphatase activity of the microalgae *Chlorella vulgaris* on two gold interdigitated electrodes	Wastewater	1	nr	[42]
Colorimetry (ratio 540 nm/396 nm) of glutathione-stabilized silver nanoparticles	Standard	600	nr	[125]
Conductometric measurement of alkaline phosphatase activity in enzyme membrane on gold interdigitated electrodes	Tris-nitrate buffer	5,000	4	[62]
Nine cross-reactive sensing fluorescent elements on micro-plate & data processed by pattern recognition	Mineral waters	6,000	nr	[65]

* LoD: Limit of Detection; RSD: Relative Standard Deviation; nr: not reported; DPASV: Differential Pulse Anodic Stripping Voltammetry; SWASV: Square-Wave Anodic Stripping Voltammetry; LSASV: linear-scan anodic stripping voltammetry.

**Table 8.** Analytical characteristics of electrochemical sensors for lead determination in water, published between 2007 and 2009.

**Method**	**Tested samples**	**LoD (μg/L)**	**RSD (%)**	**Ref.**
DPASV on multiwalled carbon nanotubes/Bi film modified glassy carbon electrode	Tap & river water	0.1	4.1	[34]
Potentiometry on 5,5,dithiobis(2-nitrobenzoic acid)/PVC membrane	Mine water	0.12	20	[128]
SWASV on hydroxyapatite modified carbon paste electrode	Tap & wastewater	0.16	4.1	[126]
SWASV on antimony film carbon paste electrode	Lake water	0.2	1.2	[40]
LASV on graphite felt electrode	Standards	0.2	15	[127]
DPASV on carbon paste electrode modified with biomolecular chitosan	Tap water	0.3	3.5	[130]
SWASV on bismuth-film electrodes	River water	0.5	4.4	[43]
DPASV on bismuth film/graphite electrode	Acetate pH 4	0.5	nr	[36]
DPASV on bismuth/poly(p-aminobenzene sulfonic acid) film electrode	Tap water	0.80	3.9	[39]
SWASV on carbon disk screen-printed electrode with Bi ions	Wastewater	0.9	7.4	[54]
Conductometric measurement of alkaline phosphatase activity of the microalgae *Chlorella vulgaris* on two gold interdigitated electrodes	Wastewater	1	nr	[42]
SWASV on polymer-modified glassy carbon electrode	Plating wastewater	1	2.5	[131]
DPASV on boron-doped diamond	Acetate buffer	1.1	nr	[35]
DPASV on glassy carbon electrode modified with *p*-tert-butylthiacalix[4]arene	Lake & tap waters	1.7	2.9	[49]
SWASV on screen-printed graphite electrode	Seawater	1.8	4.9	[52]
SWASV on bismuth nanoparticle modified boron doped diamond	Standard pH 1.2	1.9	nr	[50]
SWASV on glassy-carbon electrodes modified with Nafion & thiol monolayer composite on mesoporous supports	River & ground waters	2.7	5	[51, 132]
LASV on a zeolite NH4-Y modified carbon paste electrode	Groundwater & industrial wastewater	3.6	7	[44]
SWASV on carbon paste electrode modified with 2-aminothiazole	Tap water	4.5	10	[116]
SWASV on nitrogen doped diamond-like carbon microelectrode array	Acetate buffer pH 4	4.6	nr	[36]
Cyclic voltammetry of invertase–glucose oxidase activities on platinum microelectrode	Phosphate buffer	6.2	nr	[53]
ASV on screen-printed Bismuth oxide/graphite electrode	Wastewater	8	5.6	[57]
SWASV on bismuth electrode	Pore & groundwater	8	15	[56]
LSASV on antimony nanoparticle modified boron doped diamond	Standard pH 1	18.5	nr	[59]
Potentiometry on polyaminoanthraquinone microparticle/PVC membrane	Spiked rain & tap waters	160	nr	[133]
LASV on Nitrogen doped tetrahedral amorphous carbon thin films/silicone	Standard pH 1	210	nr	[121]
SWASV on polymer-modified glassy carbon electrode	Plating wastewater	400	2.8	[45]
Potentiometry on substituted macrocyclic diamides/PVC membrane	Standards	410		[134]
Conductometric measurement of alkaline phosphatase activity in enzyme membrane on gold interdigitated electrodes	Standards	40,000	4	[62]

* LoD: Limit of Detection; RSD: Relative Standard Deviation; nr: not reported; DPASV: Differential Pulse Anodic Stripping Voltammetry; SWASV: Square-Wave Anodic Stripping Voltammetry; ; LSASV: linear-scan anodic stripping voltammetry.

**Table 9.** Analytical characteristics of optical sensors for lead determination in water published between 2007 and 2009.

**Method**	**Tested sample**	**LoD (μg/L)**	**RSD (%)**	**Ref.**
Fluorimetry (475/518nm) on thrombin-binding aptamer probe labeled with carboxyfluorescein & 4-([4-(dimethylamino)phenyl]azo)benzoic acid (turn on)	Pond water	0.1	1.3	[90]
Fluorimetric scanning of metal dependent DNAzymes microarray	Spiked river water	2	6.7	[135]
Colorimetry (434 nm) of 4-hydroxysalophen on cellulose membrane	River & wastewater	18	2.1	[136]
Fluorimetry (365/418 nm) on polyfluorene with two benzo-18-crown-6 side chains (turn off)	Standards	1,000	nr	[137]
Colorimetry (530 nm) on diphenylcarbazone/PVC membrane	Natural & wastewater	1,300	nr	[138]

* LoD: Limit of Detection; RSD: Relative Standard Deviation; nr: not reported; DPASV: Differential Pulse Anodic Stripping Voltammetry; SWASV: Square-Wave Anodic Stripping Voltammetry; LSASV: linear-scan anodic stripping voltammetry.

**Table 10.** Summary of the superior limit concentrations (μg/L) in organic substances, recommended or statutory in freshwater.

	**Superficial Water**		**Drinking Water**	

	**WFD [2] AA-EQS**	**SEQ-Eaux [32]**	**Order in Council 2001-1220 [33]**	**SEQ Underground water**

Benzene	10	0.5	1.0	0.5
Chloroform	2.5	1.2	100	5
Atrazine	0.6	0.02	0.1	0.05
Isoproturon	0.3	0.02	0.1	0.05
Chlorfenvinphos	0.1	0.0003	0.1	0.05
Chlorpyrifos	0.03	0.00005	0.1	0.05
DDTpp’	0.01	0.0002	0.1	0.05

* AA-EQS: Environmental quality standards expressed as an annual average value. SEQ-Eau : Système d’Evaluation de la Qualité de l’eau (Water Quality Evaluation System) developed by French Water Agencies.

**Table 11.** Micro-sensors for organophosphate and carbamates (in italics) determination in water. WFD priority organic substance in bold type.

**Method**	**Compound**	**LoD**	**Ref.**
Colorimetry on gold nanoparticles	Chlorpyrifos Malathion	20 μg/L 100 μg/L	[146]
Guided shear horizontal surface acoustic wave devices on LiTaO_3_	Phosmet Parathion	5 mg/L 2 mg/L	[149]
Voltammetry on clay modified electrodes containing Ni_2_Al-NO_3_	Glyphosate Glufosinate	169 μg/L 905 μg/L	[150]
Amperometric activity measurement of acetylcholinesterase immobilized on screen-printed graphite electrode	Chlorpyrifos	2 μg/L	[151,152]
Voltammetry on glassy carbon electrode modified with a poly-L-cysteine film	Methyl parathion	1.7 μg/L	[153]
Amperometric activity measurement of acetylcholinasterase immobilized on gold nanoparticles and silk fibroin modified platinum electrode	Methyl paraoxon *Carbofura* Phoxim	6 ng/L 7 ng/L 0.6 μg/L	[148]
Amperometric activity measurement of acetylcholinesterase immobilized on calcium carbonate-chitosan composite film	Methyl parathion	1 μg/L	[154]
Amperometric activity measurement of acetylcholinesterase and choline oxidase within separate hybrid mesoporous silica membranes	Diazinon-oxon	0.37 μg/L	[155]
Amperometric activity measurement of acetylcholinesterase on gold-platinum bimetallic nanoparticles onto 3-amino-propyltriethoxy silane modified glassy carbon electrode	Paraoxon ethyl Sarin *Aldicarb*	9.5 μg/L 6 μg/L 48 μg/L	[156]
Amperometric activity measurement of acetylcholinesterase on poly(dimethosiloxane)-poly(diallydimethylammonium)/gold nanoparticles composite film	Paraoxon Parathion	0.5 ng/L 1.0 ng/L	[147]
Voltammetry on nano-alumine film modified electrode	Parathion	0.3 μg/L	[157]
Amperometric reactivation measurement of cholinesterase from organophosphorus-inhibited rat saliva on carbon nanotube modified screen printed carbon electrode	Paraoxon	0.14 μg/L	[158]

* LoD: Limit of Detection.

**Table 12.** Micro-sensors for pesticide determination in water, other than organophosphate and carbamates. WFD priority organic substances in bold type.

**Method**	**Compound**	**LoD**	**Ref.**
Voltammetry on conducting MIP deposited on an platinum electrode	**Atrazine**	4.5 μg/L	[140]
Surface plasmon resonance (SPR) on monoclonal anti-bodies immobilized on a gold sensor surface	**Atrazine**	20 ng/L	[141]
Oxygen production and fluorescence measures on the algae *Elodea canadensis* culture	**Atrazine**	10 μg/L	[142]
Surface plasmon resonance (SPR) on monoclonal anti-bodies immobilized on sensor chip surface	**Isoproturon**	0.1 μg/L	[143]
Resonance characteristics hepatoma cultured on an indium tin oxide surface of quartz crystal	Paraquat	2.7 mg/L	[144]
Surface plasmon resonance (SPR) on monoclonal antibodies on gold-thin layer	**DDT**	15 ng/L	[145]

* LoD: Limit of Detection.

**Table 13.** Micro-sensors for solvent determination in water.

**Method**	**Compound**	**LoD**	**Ref.**
Infrared spectroscopy on polydimethylsiloxane (PDMS) membrane	Benzene	0.6 mg/L	[159]
Mid-infrared spectroscopy on 2-(2-hydroxy-5-tert-octylphenyl)benzotriazol/PVC membrane	Benzene	5.0 mg/L	[160]
Impedance spectrometry on sensor array made up with electrodeposited polythiophene films onto interdigitated gold electrodes	Chloroform	0.1 mg/L	[161]

* LoD: Limit of Detection
